# Ether

**Published:** 1862-12

**Authors:** J. Bouisson

**Affiliations:** Montpelier


ETHER.—*Letter from M. Bouisson, of Montpelier, France.* Dr. Hodge read the following answer to the circular issued by the Ether Committee, which was just received, and which, he remarked, seemed worthy of notice, coming as it does from one of the earliest students of the subject of anæsthesia. M. Bouisson, soon after the general introduction of anæsthetic agents, published a large and elaborate memoir on their uses and effects, which to this day remains one of the most valuable we possess. Surrounded by influences calculated to prejudice him in favor of chloroform, it is pleasant to find a French surgeon acknowledging the perfect security of ether, even though inclined occasionally to incur the dangers of an agent the treachery of which he recognizes and of whose frequently fatal effects he is fully aware.

MONTPELIER, June 25th, 1862.

"I have myself never seen any case in which sulphuric ether was productive of injury. After sixteen years of hospital practice, I persist in the opinion that anæsthesia effected by sulphuric ether is of a value which it is requitable to lose by the use of chloroform alone. In availing myself of anæsthesia, I have adopted an eclectic practice, making use of ether in all these cases where I have reason to fear the insidious activity of chloroform. The necrology of the latter agent is, unfortunately, already overfreighted, and I am afraid in employing it exclusively, the risk is run of depreciating the use of all anæsthetics. Such a risk can not be alleged against ether, and although slow in its effects, the security which belongs to its exhibition ought to preserve its use in the practice of surgery. J. BOUISSON.

